# Capturing cooperative interactions with the PSI-MI format

**DOI:** 10.1093/database/bat066

**Published:** 2013-09-24

**Authors:** Kim Van Roey, Sandra Orchard, Samuel Kerrien, Marine Dumousseau, Sylvie Ricard-Blum, Henning Hermjakob, Toby J. Gibson

**Affiliations:** ^1^Structural and Computational Biology Unit, European Molecular Biology Laboratory (EMBL), Meyerhofstrasse 1, D-69117 Heidelberg, Germany, ^2^Proteomics Services, EMBL Outstation, European Bioinformatics Institute (EBI), Wellcome Trust Genome Campus, Hinxton, Cambridge, CB10 1SD, UK and ^3^UMR 5086 CNRS - Université Lyon 1, Institut de Biologie et Chimie des Protéines, 7 passage du Vercors, 69367 Lyon Cedex 07, France

## Abstract

The complex biological processes that control cellular function are mediated by intricate networks of molecular interactions. Accumulating evidence indicates that these interactions are often interdependent, thus acting cooperatively. Cooperative interactions are prevalent in and indispensible for reliable and robust control of cell regulation, as they underlie the conditional decision-making capability of large regulatory complexes. Despite an increased focus on experimental elucidation of the molecular details of cooperative binding events, as evidenced by their growing occurrence in literature, they are currently lacking from the main bioinformatics resources. One of the contributing factors to this deficiency is the lack of a computer-readable standard representation and exchange format for cooperative interaction data. To tackle this shortcoming, we added functionality to the widely used PSI-MI interchange format for molecular interaction data by defining new controlled vocabulary terms that allow annotation of different aspects of cooperativity without making structural changes to the underlying XML schema. As a result, we are able to capture cooperative interaction data in a structured format that is backward compatible with PSI-MI–based data and applications. This will facilitate the storage, exchange and analysis of cooperative interaction data, which in turn will advance experimental research on this fundamental principle in biology.

**Database URL:** http://psi-mi-cooperativeinteractions.embl.de/

## Introduction

Cells are subject to ever changing environmental or cell state-specific conditions, and must thus continuously monitor and integrate a wide variety of external and internal signals to generate appropriate responses. The complex biological processes that mediate cell regulation and signalling are effected by intricate and interlinked molecular interaction networks that are tightly controlled by modulating the binding properties of the constituting molecules, which is achieved by the interplay between their abundance, subcellular localization, modification state and interactions with other components (1–3). These networks control context-dependent assembly of large dynamic macromolecular ensembles that can perform a wide range of biological functions by operating as signalling machines that make regulatory decisions to drive signal propagation and elicit cellular responses (4–6). Because the subunits of such an assembly regularly influence each other’s function, resulting in an altered catalytic or binding activity, the distinct binding events between these components are often not independent. Instead, many interactions are cooperative, affecting each other positively or negatively (7, 8). Owing to these interdependencies, such a system is characterized by abrupt transitions between the active and inactive states in response to changes in its environment (8, 9). Cooperative interactions are essential for cell biology, as they govern the dynamic and context-dependent nature of cell signalling by conditionally regulating molecular interactions and biochemical reactions, and thereby dictate the switch-like behaviour of regulatory complexes (3, 5, 7, 8). As such, they mediate regulatory decision-making, allow integration of multiple signals and contribute to the robustness of cell regulatory systems, a key property enabling these systems to maintain desired characteristics despite stochastic fluctuations in the behaviour of their parts or environment (3, 5, 10, 11).

The protagonists of cell regulation, protein, RNA and DNA molecules have inherent properties that facilitate cooperative binding. Firstly, these biopolymers can occur in multiple conformations that can have distinct functionality, with the predominant conformer depending on the molecule’s environment, for instance, the presence of a particular binding partner (12–16). Secondly, they have a modular architecture, containing discrete functional units such as globular domains with catalytic or binding activities (1, 17), disordered interaction interfaces such as short linear motifs (18), transcription factor binding sites in DNA (13, 19), protein binding sites in RNA (20, 21) and sites for covalent modification (22–24). Overlapping binding interfaces promote competitive binding by engaging in mutually exclusive interactions, whereas overlapping or adjacent modification sites allow modulation of a binding interface by a modification event. Alternatively, adjacent interaction sites can mediate multivalent binding (3). These features allow the interactions of proteins, RNA or DNA to be regulated by the two basic mechanisms underlying cooperative binding: allostery, where the functional properties of a molecule at one site are altered by a perturbation at a distinct site (7, 25), or pre-assembly, where pre-formation of a complex affects interactions of its components through different non-allosteric mechanisms (3, 5, 7).

Despite the importance of cooperative interactions in regulating biological molecular systems, they are currently not adequately captured in bioinformatics resources, for instance, interaction databases such as IntAct (26), the Database of Interacting Proteins (DIP) (27) and the Molecular Interaction database (MINT) (28), but also pathway databases like Reactome (29). Although these resources provide a large amount of useful data in great detail, the true biological complexity of the interactions and processes they describe can in many cases not be represented. Moreover, the lack of annotation of cooperativity between different binding events can even lead to misinterpretation of the data. One aspect that contributes to the discrepancies between the complexity of *in vivo* biological interactions and their *in silico* annotation is the lack of a computer-readable standard data format to represent these interdependencies in full detail. The previously defined Biomolecular Interaction Network Database (BIND) data specification enabled representation of interaction interdependencies and ordered binding events (30); however, it was never adopted as a standard molecular interaction data format.

Because several standards that capture different aspects of cell regulation and signalling are already available and actively being used (31), the development of a new data format to represent cooperative interactions would be counterproductive. Instead, it is more sensible to adapt or extend an existing molecular interaction data standard. At present, the most widely used community standard for molecular interaction data is the XML-based PSI-MI format, developed by the Proteomics Standards Initiative of the Human Proteome Organization (HUPO) (32, 33). This format is used by a variety of data resources (26–28, 34–36) and software tools (37–39) and has proven invaluable for efficient molecular interaction data exchange in a standardized manner. However, molecular interactions are captured independently from each other. In this article, we illustrate the basic mechanisms that mediate cooperative binding, which were defined based on a literature survey, and describe how the current PSI-MI XML format (version 2.5.4) can capture cooperative interaction data by using new controlled vocabulary (CV) terms that were added to the PSI-MI CV (version 2.5.5). In combination with previously defined PSI-MI CV terms, such as those used to specify the type of interaction, these new terms enable detailed description of distinct binding events, their interdependencies and the underlying molecular mechanisms. This strategy avoids having to make any structural changes to the PSI-MI XML schema, keeping it backward compatible and the existing data viable, and allowing additional annotation of cooperative effects for interactions already curated in available databases.

## Results

### Classification of cooperative interactions

Cooperative binding arises when distinct molecular interactions, including enzymatic modification of a molecule, influence each other either positively or negatively. However, there are different mechanisms through which an interaction can affect other interactions. We performed a literature survey to categorize cooperative interactions and to define the classes of information that need to be captured to comprehensively annotate them. All the examples that were collected in this study could be classified in two main categories with respect to the mechanism underlying cooperative binding: allostery and pre-assembly (5, 7, 9, 25). For each of the mechanisms discussed here, illustrated examples curated in the format presented in this article can be found on the documentation website (http://psi-mi-cooperativeinteractions.embl.de).

### Allostery

Allostery can be defined as a change in binding (k-type response) or catalytic (v-type response) properties of a biopolymer at one site by a perturbation at a distinct site, due to reciprocal energetic coupling between the two events (25). It allows fast, combinatorial and reversible regulation of molecular function and is intrinsic to the control of signal transduction and metabolism, enabling adaptation to changing conditions by managing signal transmission and metabolic pathway fluxes (25, 40). Although the concept of allostery has been established for a long time, and different models have been defined (41), it was traditionally associated with the regulation of metabolic enzymes like phosphofructokinase-1 by metabolites (42, 43) and the cooperative binding of oxygen to haemoglobin (44–46).

However, owing to advances in spectroscopic techniques that allow characterization of molecular dynamics at the level of single molecules, a new view on allostery has emerged. While an allosteric response was originally believed to result from a large structural change elicited by binding of a small molecule to a symmetric oligomeric protein, communication between two distinct binding sites on an allosteric molecule can be mediated by small structural rearrangements or a change in its dynamic properties (12, 47–51). Owing to their inherent flexibility resulting from backbone and side chain motions on a wide range of timescales, most proteins do not exist as a single stable conformation. Instead, they sample an ensemble of accessible conformations that are in dynamic equilibrium, with the lowest energy substate having the highest probability of being populated and the energy barriers between the different substates determining the timescale of switching between populations (12, 50, 51). A perturbation, such as a binding event or post-translational modification (PTM), remodels the energy landscape of a protein by stabilizing one of the conformers. This shifts the equilibrium between the pre-existing conformations, resulting in a redistribution of their relative populations. The overall behaviour of a protein, and the outcome of the processes it regulates, thus depends on the prevailing substate, which is determined by the protein’s cellular context, and can be reversibly altered by changing its surroundings to induce interconversion between the different conformers. If the different conformers have distinct functionality at a site that is distinct from the site of perturbation, allosteric behaviour is observed (12, 40, 41, 50, 51).

**Figure 1.**
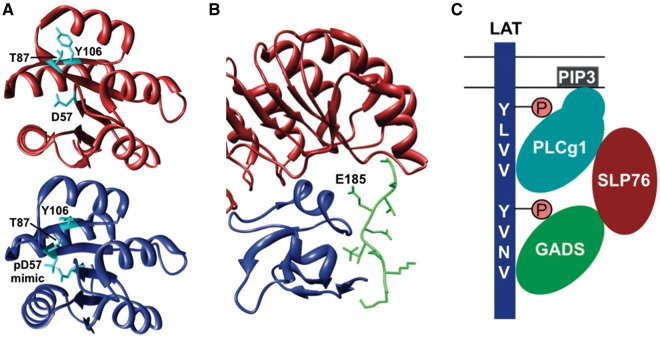
Allostery (**A**) and pre-assembly (**B**, **C**) as basic mechanisms for cooperative binding. (**A**) Phosphorylation of the D57 residue in CheY allosterically regulates binding of FliM by stabilizing the active form of CheY (blue) (PDB:1ZDM) (58), which has a higher affinity for FliM compared with the inactive form (red) (PDB:1E6K) (59) owing to burial of the Y106 residue. (**B**) Recognition of p27^Kip1^ (green) by the SCF^Skp2^ ubiquitin ligase requires pre-assembly of the Skp2 (red)-Cks1 (blue) complex because these two proteins form a continuous composite binding site required for binding of p27^Kip1^ (PDB:2AST) (60). (**C**) Cooperative binding, which results from configurational pre-organization, of the SH2 and PH domains of PLCg1 mediates recruitment to LAT by binding to phosphorylated SH2-binding motifs in LAT and to phosphoinositides in the plasma membrane, respectively. The same mechanism controls binding of other subunits to the LAT-nucleated complex, resulting in multiple discrete binding events that stabilize each other, allowing regulated assembly of a meta-stable complex (GADS: GRB2-related adapter protein 2; SLP76: Lymphocyte cytosolic protein 2; PIP3: Phosphatidylinositol-3,4,5-trisphosphate). Figures A and B were generated using UCSF Chimera (83).

This means that the function of all dynamic biopolymers can potentially be allosterically regulated, including monomeric proteins, but also DNA and RNA molecules (12, 14, 16, 52–54), and that such regulation is not only mediated by small molecules, but can also be effected by binding of another macromolecule, a modification event, or a change in the environment, for instance, pH (49, 55). Examples that illustrate the wide regulatory potential of allostery include binding of Pygopus homolog 1 (Pygo1), involved in Wnt signalling, to dimethylated histone H3 (H3K4me2). This interaction is allosterically regulated by binding of B-cell CLL/Lymphoma 9 protein (Bcl9) to a site distinct from the H3-binding pocket, resulting in stabilization of conformational rearrangements that shape Pygo1 for optimal recognition of H3K4me2 (56). Allosteric control of protein function by PTM is involved in regulating the binding of the *Escherichia coli* chemotaxis protein CheY to the flagellar motor protein FliM. The activated form of CheY, which has increased affinity for FliM owing to burial of the Y106 residue at the FliM-binding site, is stabilized by phosphorylation of the D57 residue in CheY and subsequent formation of a hydrogen bond between the phosphate moiety and the T87 residue (57) (Figure 1A).

### Pre-assembly

The second mechanism underlying cooperativity is pre-assembly, a non-allosteric mechanism where the strength of an interaction depends on whether or not a particular complex was pre-formed (3, 5, 7). There are different mechanisms through which pre-assembly of a complex can affect subsequent interactions of any of its components.

Complex formation can result in the generation of a continuous binding site that spans multiple components and is only functional in the context of the assembled complex. Such a mechanism is involved in targeting the cyclin-dependent kinase (Cdk) inhibitor p27^Kip1^ for proteasomal degradation by ubiquitylation, which is catalysed by the SCF^Skp2^ ubiquitin ligase. Recognition of p27^Kip1^ by SCF^Skp2^ not only depends on the F-box protein Skp2, but also requires association of Skp2 with the accessory protein Cks1. Skp2 and Cks1 together form a continuous composite binding site for p27^Kip1^ that spans both these proteins. Some residues of p27^Kip1^ interact with residues of Skp2, while others interact with residues of Cks1. The E185 residue of p27^Kip1^ inserts into the interface between Skp2 and Cks1 and makes contacts with both proteins. As a result, p27^Kip1^ can only be marked for degradation by ubiquitylation when Skp2 is associated with Cks1 (60) (Figure 1B).

In the context of pre-assembly, modification of a biopolymer can also be regarded as a pre-formation step, with prior binding of a modifying enzyme and concomitant modification of a binding interface often having profound effects on subsequent interactions. This regulatory mechanism is prevalent in and important for control of cell metabolism and signalling, and allows fast, reversible and context-dependent rewiring of interaction networks (3, 4, 22, 24). Owing to their structural and chemical properties, modifications can affect, to various degrees, subsequent interactions of a molecule in a non-allosteric manner by altering the physicochemical compatibility with and intrinsic specificity for its binding partners. In some cases, modification of a binding site is a prerequisite for an interaction, while in other cases modification can further enhance the strength of an interaction (61–63). Alternatively, binding site modification can also result in partial inhibition of an interaction (64), or even complete abrogation of an interaction (65). Modulation of an interaction by modification of multiple residues even allows for rheostatic control of binding strength, where the affinity for an interaction partner is gradually altered by multiple modifications that additively enhance or diminish the interaction. This allows for fine-tuned control of binding depending on the intensity or duration of a signal or the integration of multiple signals (66).

Many biopolymers contain overlapping or adjacent binding sites that engage in mutually exclusive interactions with distinct binding partners. Binding of a molecule to one such site will result in hiding of the mutually exclusive binding site by sterically blocking its accessibility and thereby inhibit binding of the competing molecule. The outcome of the competition depends on the intrinsic specificity and affinity of the interaction interface for the different binding partners as well as the local abundance of these competitors. While the former can be modulated by modification, as discussed previously, the latter can be controlled by scaffolding or by altering the expression level, stability or subcellular localization of the competing binding partners (3). An extreme case of competitive binding occurs when there is a large difference in affinity for or local abundance of the different competitors, as binding of the high-affinity or abundant partner will preclude the second partner from binding (3, 67, 68).

A final mechanism through which pre-assembly of a complex can mediate cooperative interactions is configurational pre-organization, which involves distinct binding sites on multivalent ligands that can form multiple discrete interactions with one or more binding partners (5, 7). An initial binding event at one site pre-organizes other sites, thereby reducing their degrees of freedom, which reduces the entropic costs of their interaction (7). In addition, the local concentration of the binding interfaces near their target site is increased, which promotes their interaction (40). Also, the combined strength of multiple interactions increases the enthalpic stability of each interaction, a phenomenon known as the avidity effect (7). For instance, recruitment of the phospholipase PLCg1 to the Linker for Activation of T-cells (LAT) adaptor protein, involved in T-cell signalling, depends on multiple discrete binding sites in PLCg1. Its Src Homology 2 (SH2) domain binds to phosphorylated LAT, while its Pleckstrin Homology (PH) domain interacts with phosphoinositides in the plasma membrane. Either of these two interactions will pre-organize the other domain for binding and thereby facilitate its interaction. Both discrete binding events will then stabilize each other and increase the enthalpic stability of the complex (69) (Figure 1C). This mechanism generally mediates the assembly of meta-stable signalling platforms whose subunits associate with higher affinity than the sum of the individual interactions, which usually are relatively weak (5).

### The PSI-MI format

The PSI-MI data standard was developed to facilitate the exchange, comparison and verification of molecular interaction data (32, 33). Use of the PSI-MI XML schema ensures that all molecular interaction data representations are consistent in form. To ensure consistency in content, an extensive list of CV terms has been defined (32, 33). In addition, a community guideline called the Minimum Information about a Molecular Interaction Experiment (MIMIx) has been defined to advise users on how to describe a molecular interaction experiment and specify the minimum information that is required to unambiguously report an interaction (70). The XML schema, CV terms and MIMIx guidelines can be found at the HUPO PSI-MI website (http://www.psidev.info/groups/molecular-interactions).

**Figure 2.**
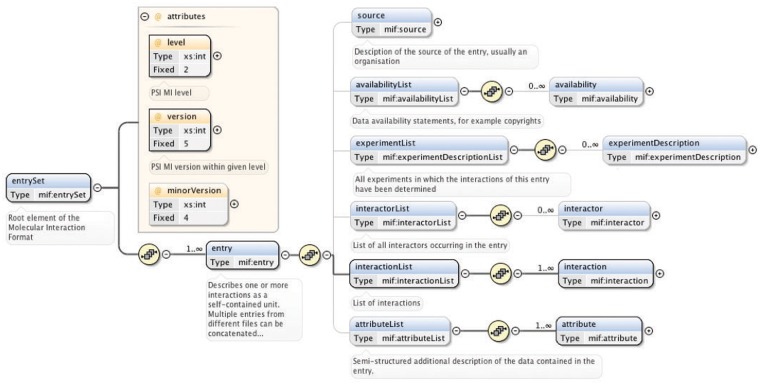
Main elements of the PSI-MI XML schema (version 2.5.4). The entrySet root element of the schema contains one or more entry elements that describe one or more interactions within its six main child elements. These six elements have additional child elements that allow detailed annotation of experimental interaction data and metadata. A plus sign within a circle denotes an element has been collapsed. Blue and yellow boxes indicate elements and attributes of an element, respectively. Bold connections are used for required elements and attributes. All compositors (yellow circles) in the figure indicate an ordered sequence of contained particles. This figure is based on (33) and generated using the oXygen XML editor.

The ‘entrySet’ root element of the PSI-MI XML schema contains one or more ‘entry’ elements, each of which describe one or more interactions together with all associated data as a self-contained unit (33) (Figure 2). Comprehensive and thorough annotation of experimental interaction data and metadata is covered by the six child elements of the ‘entry’ element: ‘source’ (the source of the data, for instance, an organization), ‘availabilityList’ (the availability of the data, for instance, copyrights), ‘experimentList’ (the experiments used to determine the interactions, for instance, coimmunoprecipitation), ‘interactorList’ (the identity of the interacting molecules), ‘interactionList’ (the interactions between a set of molecules) and ‘attributeList’ (semi-structured additional information on the data) (33) (Figure 2).

### Describing cooperative interactions in the PSI-MI format

Although the PSI-MI data specification does not intrinsically capture interdependencies between distinct binding events, we defined a new set of CV terms to enable the representation of cooperative interactions using the current version of the format (Tables 1 and 2). As this strategy avoids making structural changes to the XML schema, backward compatibility is maintained. To illustrate how such interactions can be captured in the PSI-MI format, data from several publications studying the molecular mechanisms involved in the phosphorylation of substrates, in this case Cell division control protein 6 homolog (Cdc6), by the Cyclin A-Cdk2 complex were collected, and the interdependencies between the different binding events that are involved were annotated in a PSI-MI XML file. The complete XML file and an HTML rendering of this file are included in the supplementary material (Supplementary Figures S1 and S2).

**Table 1.** Types of cooperative interaction data that can be annotated in the PSI-MI format

Type of data	Interaction attribute names	Interaction attribute value
General data		
The mechanism underlying cooperative binding	Allostery (MI:1157)	None
	Pre-assembly (MI:1158)	
The interaction that is influenced	Affected interaction (MI:1150)	Interaction ID
Whether the affected interaction is augmented or diminished	Positive cooperative effect (MI:1154)	None
	Negative cooperative effect (MI:1155)	
Quantification of the cooperative effect	Cooperative effect value (MI:1152)	Fold change of an interaction parameter in the absence versus presence of a perturbation
When allostery is the underlying mechanism		
The molecule that is allosterically regulated	Allosteric molecule (MI:1159)	Participant ID
The ligand that elicits the allosteric response	Allosteric effector (MI:1160)	Participant ID
The PTM that elicits the allosteric response	Allosteric PTM (MI:1175)	Feature ID
The type of allosteric response	Allosteric k-type response (MI:1162)	None
	Allosteric v-type response (MI:1163)	
The mechanism that mediates the allosteric response	Allosteric change in structure (MI:1165)	None
	Allosteric change in dynamics (MI:1166)	
The type of allostery	Homotropic allostery (MI:1169)	None
	Heterotropic allostery (MI:1168)	
When pre-assembly is the underlying mechanism		
The type of pre-assembly response	Composite binding site formation (MI:1171)	None
	Altered physicochemical compatibility (MI:1172)	
	Binding site hiding (MI:1173) Configurational pre-organization (MI:1174)	

This table lists the types of data that can be described in a PSI-MI XML file using the new cooperative interaction-specific PSI-MI CV terms. The middle and right columns indicate what CV terms to use to annotate a particular data type and, when applicable, what value to use for an interaction attribute named with a particular CV term, respectively.

**Table 2.** New cooperative interaction-specific PSI-MI CV terms

Term name	Term id	Relationships	Definition
Cooperative interaction	MI:1149	part_of MI:0000! molecular interaction	A set of molecular binding events that influence each other either positively or negatively through allostery or pre-assembly. In this context, covalent PTMs are considered as binding events. CV terms that are part of this term allow the description of cooperative interactions using the current PSI-MI schema.
Affected interaction	MI:1150	is_a: MI:0664! interaction attribute name	For an interaction that has a cooperative effect on a subsequent interaction, this term indicates which subsequent interaction is affected. The affected interaction is identified by referring to its interaction id.
		part_of MI:1149! cooperative interaction	
Participant-ref	MI:1151	is_a: MI:0668! feature attribute name	Referring to a previously described interaction as a participant allows the description of ordered assembly of molecular complexes in PSI-MI2.5. When one of the components of the preformed complex has a feature, the participant-ref term indicates on which component this feature is located. The component is identified by referring to its participant id in the previous interaction.
Cooperative effect value	MI:1152	is_a: MI:0664! interaction attribute name	This value quantifies the cooperative effect of an interaction on a subsequent interaction. It is the fold change of the affinity or a catalytic parameter of a molecule for one ligand in the absence, versus presence, of a second ligand or a PTM.
		part_of MI:1149! cooperative interaction	
Cooperative effect outcome	MI:1153	part_of MI:1149! cooperative interaction	For an interaction that has a cooperative effect on a subsequent interaction, this term indicates whether this effect is positive or negative, i.e. whether the subsequent interaction is augmented or diminished.
Positive cooperative effect	MI:1154	is_a: MI:0664! interaction attribute name	This term specifies that an interaction augments a subsequent interaction.
		is_a: MI:1153! cooperative effect outcome	
Negative cooperative effect	MI:1155	is_a: MI:0664! interaction attribute name	This term specifies that an interaction diminishes a subsequent interaction.
		is_a: MI:1153! cooperative effect outcome	
Cooperative mechanism	MI:1156	part_of MI:1149! cooperative interaction	For an interaction that has a cooperative effect on a subsequent interaction, this term indicates the process that mediates this effect.
Allostery	MI:1157	is_a: MI:0664 interaction attribute name	Reciprocal energetic coupling between two binding events at distinct sites on the same molecule. The first binding event alters the binding or catalytic properties of the molecule for the second binding event.
		is_a: MI:1156! cooperative mechanism	
Pre-assembly	MI:1158	is_a: MI:0664! interaction attribute name	A non-allosteric mechanism where the strength of an interaction depends on whether or not a particular molecular complex already exists.
		is_a: MI:1156! cooperative mechanism	
Allosteric molecule	MI:1159	is_a: MI:0500! biological role	A molecule whose binding or catalytic properties at one site are altered by allosteric PTM or binding of an allosteric effector at a distinct site. An allosteric molecule is identified by referring to its participant id.
		is_a: MI:0664! interaction attribute name	
		part_of MI:1149! cooperative interaction	
Allosteric effector	MI:1160	is_a: MI:0500! biological role	A ligand that elicits an allosteric response on binding to a target molecule.
		is_a: MI:0664! interaction attribute name	
		part_of MI:1149! cooperative interaction	
Allosteric response	MI:1161	part_of MI:1149! cooperative interaction	This term describes the effect of an allosteric binding event. It specifies which properties of the allosteric molecule are altered, i.e. whether the interaction alters either (a) binding or (b) catalytic properties of the allosteric molecule at a site distinct from the allosteric site.
Allosteric k-type response	MI:1162	is_a: MI:0664! interaction attribute name	An allosteric response in which the affinity of a molecule is altered.
		is_a: MI:1161! allosteric response	
Allosteric v-type response	MI:1163	is_a: MI:0664! interaction attribute name	An allosteric response in which catalysis (kcat or Vmax) of an enzyme is altered.
		is_a: MI:1161! allosteric response	
Allosteric mechanism	MI:1164	part_of MI:1149! cooperative interaction	The process that mediates the allosteric response of a molecule on allosteric PTM or binding of an allosteric effector.
Allosteric change in structure	MI:1165	is_a: MI:0664! interaction attribute name	The allosteric mechanism where changes in the local structure of an allosteric molecule result in altered binding or catalytic properties.
		is_a: MI:1164! allosteric mechanism	
Allosteric change in dynamics	MI:1166	is_a: MI:0664! interaction attribute name	The allosteric mechanism where changes in the local dynamics of an allosteric molecule result in altered binding or catalytic properties.
		is_a: MI:1164! allosteric mechanism	
Allostery type	MI:1167	part_of MI:1149! cooperative interaction	This term indicates the chemical relationship between the two ligands whose binding is allosterically coupled.
Heterotropic allostery	MI:1168	is_a: MI:0664! interaction attribute name	The type of allostery that occurs when the two ligands whose binding is allosterically coupled are not chemically identical.
		is_a: MI:1167! allostery type	
Homotropic allostery	MI:1169	is_a: MI:0664! interaction attribute name	The type of allostery that occurs when the two ligands whose binding is allosterically coupled are chemically identical.
		is_a: MI:1167! allostery type	
Pre-assembly response	MI:1170	part_of MI:1149! cooperative interaction	This term describes the way in which preformation of a molecular complex has a non-allosteric cooperative effect on subsequent interactions of its components.
Composite binding site formation	MI:1171	is_a: MI:0664! interaction attribute name	The preformation of a complex results in the generation of a continuous binding site that spans more than one component of this complex. The functional binding site does not exist outside the context of the preformed complex.
		is_a: MI:1170! pre-assembly response	
Altered physicochemical compatibility	MI:1172	is_a: MI:0664! interaction attribute name	The addition of a PTM to an interaction interface affects the physicochemical compatibility of the binding site with its binding partner. This can either induce or enhance an interaction, or result in inhibition or even abrogation of an interaction. Multisite modification can mediate rheostatic regulation of the interaction.
		is_a: MI:1170! pre-assembly response	
Binding site hiding	MI:1173	is_a: MI:0664! interaction attribute name	The occurrence of overlapping or adjacent, mutually exclusive binding sites promotes competitive binding. When there is a large difference in affinity of the different sites or in local abundance of competitors, binding at one site results in hiding of the second site, thereby precluding it from interacting when the hiding molecule is present.
		is_a: MI:1170! pre-assembly response	
Configurational pre-organization	MI:1174	is_a: MI:0664! interaction attribute name	Multivalent ligands form multiple discrete interactions with one or more binding partners. An initial binding event can pre-organize other sites for binding. This reduces the degrees of freedom of these sites, thus reducing the entropic costs of their interactions. In addition, the combined strength of multiple interactions increases the enthalpic stability of each interaction (avidity effect). As a result of such effects, interactions of this kind can have a cooperative effect on subsequent interactions.
		is_a: MI:1170! pre-assembly response	
Allosteric PTM	MI:1175	is_a: MI:0252! biological feature	A PTM that elicits an allosteric response on addition to a target molecule. An allosteric PTM is identified by referring to its feature id.
		is_a: MI:0664! interaction attribute name	
		part_of MI:1149! cooperative interaction	

This table lists the new CV terms that allow description of cooperative interactions in the PSI-MI standard data exchange format (version 2.5.4), showing their name, PSI-MI CV id, relations within the CV ontology and definition.

### Molecular mechanisms

**Figure 3.**
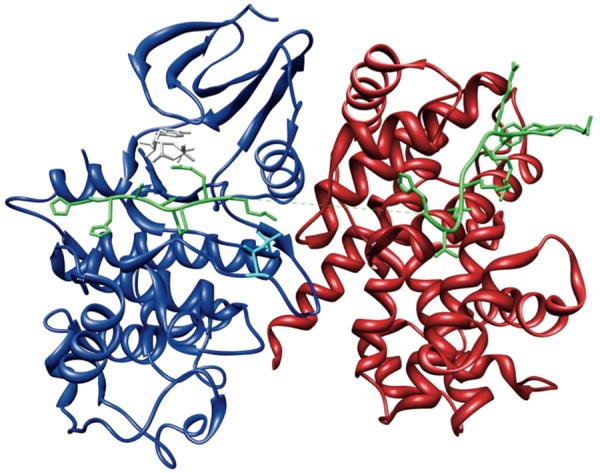
The Cyclin A-Cdk2-Cdc6 complex. Crystal structure of Cdc6 peptide bound to the active Cyclin A-Cdk2 complex (PDB:2CCI) (71), showing Cdc6 (green), Cyclin A (red) and Cdk2 (blue) phosphorylated at T160 (cyan) and bound with the ATP analogue Adenylyl Imidodiphosphate (AMPPNP) (grey). This figure was generated using UCSF Chimera (83).

**Figure 4.**
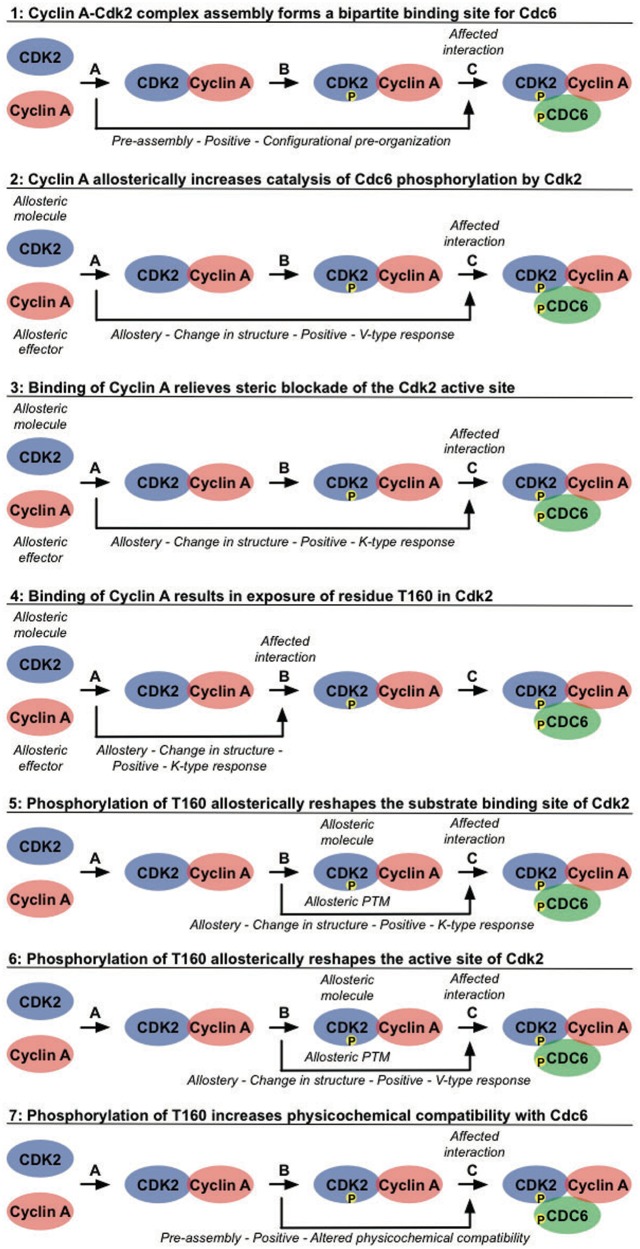
Cooperative interactions mediate recruitment and phosphorylation of Cdc6 by the Cyclin A-Cdk2 complex. The hierarchical build-up of the active Cyclin A-Cdk2 complex, phosphorylated on Cdk2, and subsequent recruitment and phosphorylation of the substrate Cdc6 is mediated by an ordered sequence of binding events that affect each other through allostery and pre-assembly. Binding of Cyclin A to Cdk2 (Interaction A) promotes binding of Cdc6 (Cooperative effects 1 and 3), catalysis of Cdc6 phosphorylation by Cdk2 (Cooperative effect 2) and phosphorylation of Cdk2 by Cdk7 (Cooperative effect 4). Phosphorylation of Cyclin A-bound Cdk2 by Cdk7 (Interaction B, Cdk7 is not shown in this figure for simplicity) also increases binding (Cooperative effects 5 and 7) and phosphorylation (Cooperative effect 6) of Cdc6 (Interaction C). See the text for more detailed descriptions. For each of these effects, the types of data that can be annotated in a PSI-MI XML file are noted in italic.

Phosphorylation of Cdc6 by Cyclin A-Cdk2 requires sequential build-up of the Cyclin A-Cdk2-Cdc6 complex (Figure 3). Distinct binding events occur in an ordered manner and cooperate to mediate the assembly of the active Cyclin A-Cdk2 complex and subsequent docking and phosphorylation of Cdc6 (Figure 4). Binding of Cyclin A to Cdk2 (Interaction A in Figure 4) pre-organizes a bipartite binding site for Cdc6, with one site on Cyclin A and one site on Cdk2 (Figure 4.1) (71). In addition, this interaction elicits several allosteric responses in Cdk2. Positional rearrangements in Cdk2 result in proper alignment of crucial active site residues that are involved in ATP orientation and magnesium coordination. This increases the efficiency of ATP binding and hydrolysis, which promotes the catalysis of substrate phosphorylation (Figure 4.2) (72, 73). Structural changes in the T loop of Cdk2 reposition this region away from the entrance of the catalytic cleft, thereby relieving steric blockade of the active site, thus allowing access for the substrate (Figure 4.3). In addition, the T160 residue, which is buried in the catalytic cleft in free Cdk2, becomes exposed and accessible for phosphorylation (Figure 4.4) (72, 73). Subsequent phosphorylation of T160 in Cdk2 by Cdk7 (Interaction B in Figure 4) results in conformational changes that make a binding pocket accessible for the proline in the P+1 position of the substrate (Figure 4.5) (73, 74). Additional changes in the active site enhance the rate of phosphotransfer by stabilizing the substrate in the transition state, thus enhancing the reactivity of Cdc6 (Figure 4.6) (73–75). Finally, this modification increases the physicochemical compatibility of Cdk2 with Cdc6, owing to direct hydrogen bonds being formed between the phosphate moiety and the lysine residue in the P+3 position in the substrate (Figure 4.7) (71, 74). These cooperative interactions mediate assembly of an active Cyclin A-Cdk2 complex in a context-dependent manner, provide substrate specificity and allow tight regulation of Cdc6 function by phosphorylation (Interaction C in Figure 4) (71).

### Representation in the PSI-MI format

Using the new CV terms we defined (Tables 1 and 2), the interdependencies between the interactions that mediate phosphorylation of Cdc6 by Cyclin A-Cdk2 can be captured in the PSI-MI format to reflect the complexity involved in regulation of such molecular systems. Based on the molecular mechanisms described above, different cooperative effects between distinct binding events can be defined, as shown in Figure 4. Briefly, binding of Cyclin A to Cdk2 (Interaction A in Figure 4, ‘CyclinA-Cdk2’ in the XML/HTML files) facilitates phosphorylation of Cdk2 by Cdk7 (Interaction B in Figure 4, ‘CyclinA_Cdk2-Cdk7’ in the XML/HTML files) (Figure 4.4) and promotes binding and phosphorylation of Cdc6 (Interaction C in Figure 4, ‘CyclinA_pCdk2-Cdc6’ in the XML/HTML files) through distinct mechanisms (Figure 4.1–4.3). Subsequent phosphorylation of Cyclin A-bound Cdk2 by Cdk7 also has several positive effects on Cdc6 recruitment and phosphorylation (Figure 4.5–4.7). To properly capture such cooperative interaction data, three important aspects need to be covered: interdependency between molecular interactions through cooperative mechanisms, complex assembly and experimental evidence for the interdependencies. To illustrate how this can be done in the PSI-MI format, the annotation of the positive effect of Cdk2 phosphorylation by Cdk7 on the catalysis of Cdc6 phosphorylation will be used as an example (Figure 4.6).

#### Interdependency between molecular interactions

**Figure 5.**
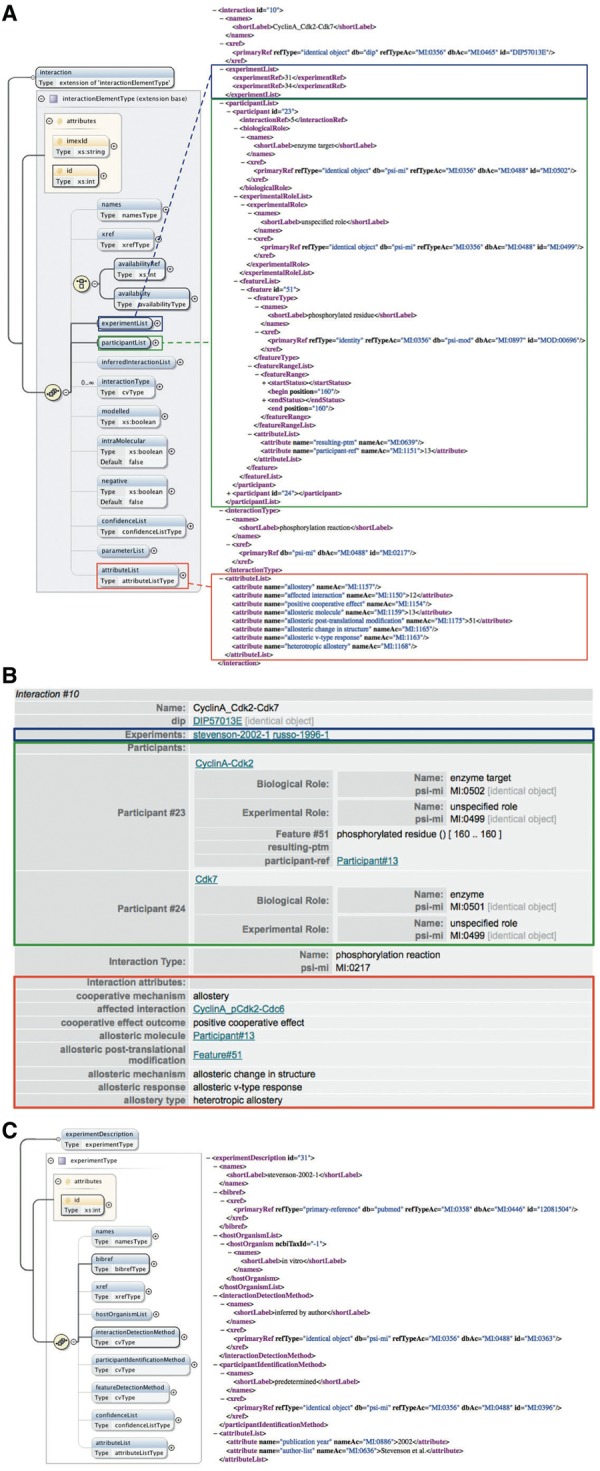
Annotation of cooperative interactions in the PSI-MI XML format. (**A**) The interaction element in the PSI-MI XML schema (left) (generated using oXygen XML editor) and one interaction involved in the phosphorylation of Cdc6 by the Cyclin A-Cdk2 complex as annotated in the PSI-MI XML file (right). The experimentList, participantList and attributeList elements of the interaction element are indicated by blue, green and red boxes, respectively. Some elements (preceded by a plus sign) are collapsed for simplicity. (**B**) HTML rendering of the same interaction shown in the PSI-MI XML file. (**C**) The experimentDescription element in the PSI-MI XML schema (left) (generated using oXygen XML editor) and one experiment providing evidence for the cooperative effect of phosphorylation of Cdk2 on the catalysis of Cdc6 phosphorylation by Cdk2 as annotated in the PSI-MI XML file (right).

Interdependencies between distinct binding events are captured in the optional ‘attributeList’ element within the ‘interaction’ element, which allows for additional description of the interaction data in a semi-structured manner (Figure 5A and B, red boxes). Each ‘attribute’ in an ‘attributeList’ can contain a value of the type string and is specified by a name, which is required, and the name accession, which is optional. The latter enables control of the ‘attribute’ name by referring to an external CV. After having established the types of data we wanted to capture, we defined new CV terms that can be used as interaction attribute names in the PSI-MI format to link molecular interactions that affect each other and annotate different aspects of their cooperative behaviour (Tables 1 and 2, Figure 4). While some of these new terms reflect the type of data that can be described for cooperative interactions, their child terms, which are also children of the ‘interaction attribute name’ term (MI:0664), can be used to annotate the data that is applicable for a specific interaction (Tables 1 and 2). Also, while attributes named by some of these new terms do not expect a value, others are meaningless without (Table 1).

Figure 5 illustrates the use of these new CV terms in a PSI-MI XML file. Phosphorylation of T160 in Cdk2 positively affects catalysis of Cdc6 phosphorylation (Figure 4.6). Using the interaction attribute names we defined, this effect is described in the ‘attributeList’ of the interaction that exerts the cooperative effect, i.e. phosphorylation of Cdk2 by Cdk7 (Figure 5A and B, red boxes). For instance, we defined the ‘cooperative mechanism’ (MI:1156) term as the parent term for the different mechanisms that can mediate cooperative binding. Its two child terms, ‘allostery’ (MI:1157) and ‘pre-assembly’ (MI:1158), can be used to specify the actual mechanism that mediates a particular cooperative effect (Table 1). Because in this example the underlying mechanism is allostery, the CV term that corresponds to this mechanism (MI:1157, ‘allostery’) is used to name an interaction attribute. Note that the attribute named by this term does not have a value between the tags. In contrast, the attribute named by the ‘affected interaction’ (MI:1150) term, which indicates the interaction that is influenced by this cooperative effect, needs the ID of the interaction that is affected (which is 12 in this case) as a value to be relevant. Other aspects that describe this effect are annotated in a similar manner (Figure 5A and B, red boxes). Because only one cooperative effect can be described in the ‘attributeList’ element of an interaction, an interaction that has more than one effect on one or more other binding events has to be repeated for each of these effects.

#### Complex assembly

Ordered assembly of molecular complexes can already be described in the current PSI-MI format by referring to a previously described interaction as a participant of a subsequent interaction (33). This is again illustrated by the phosphorylation of Cdk2 by Cdk7, which preferentially occurs when Cdk2 is bound to Cyclin A. This interaction has two participants that are described in its ‘participantList’ element (Figure 5A and B, green boxes). One of the participants is Cdk7. Because Cdk7 was already described as an interactor in the ‘interactorList’, this participant is annotated here by referencing the corresponding interactor, using its unique ID (which is 3 in this case). The second participant is the Cyclin A-Cdk2 complex. Instead of referring to a previously annotated single interactor, this participant references the interaction that describes binding of Cyclin A to Cdk2 by using the ID of this interaction (which is 5 in this case) as value of the ‘interactionRef’ element. Using interactions as participants allows representation of sequential binding events and can indicate the requirement of a prior interaction for a subsequent interaction.

If one of the complex subunits has a feature, for instance, a PTM, that is important to describe the interaction, it should be possible to discriminate between the different subunits of the complex and specify on which subunit the feature is located. To this end, a new CV term called ‘participant-ref’ (MI:1151) was defined as a child term of the ‘feature attribute name’ (MI:0668) term (Table 2). Its use is again illustrated by the phosphorylation of the Cyclin A-Cdk2 complex by Cdk7. The resulting PTM is annotated as a feature of the participant that is phosphorylated. While this participant refers to the Cyclin A-Cdk2 complex, the ‘participant-ref’ term specifies that the phosphorylated residue is located on the Cdk2 subunit of this complex (Figure 5A and B, green boxes). Within the ‘attributeList’ element of the feature, this term names an attribute that refers to Cdk2 by using the participant ID of Cdk2 (which is 13 in this case) in the interaction that describes the assembly of the Cyclin A-Cdk2 complex (the interaction with ID = ‘5’).

#### Experimental evidence

Annotation of the experimental evidence for cooperative effects exerted by an interaction is kept separate from the annotation of the methods used to determine the interaction, as combining both aspects in one entry would make it unnecessarily complicated. For example, the experimental methods used to determine the phosphorylation of Cdk2 by Cdk7 are not annotated in the PSI-MI XML file that describes cooperative assembly of the Cyclin A-Cdk2-Cdc6 complex. Instead, the single interaction is fully described in a different PSI-MI XML file that can be stored in an external resource. Using the ‘xref’ element of the interaction, these data are cross-referenced by specifying the name of the external resource, in this case DIP (27), and the primary identifier of the interaction in that resource (in this case DIP57013E) (Figure 5A and B). In the file shown in Figure 5, we instead describe the cooperative effect that phosphorylation of Cdk2 has on a subsequent interaction, and annotate the experimental evidence for this cooperativity by referring to the corresponding experiments in the entry’s ‘experimentList’ (Figure 5A and B, blue boxes). Because cooperative interactions are usually not fully described by a single experiment, and often not even in a single publication, the ‘interactionDetectionMethod’ element within an ‘experimentDescription’ element does not get a specific method assigned as a value. Instead the CV terms ‘inferred by author’ (MI:0363) or ‘inferred by curator’ (MI:0364) are used to indicate that the cooperative nature of the interactions was inferred in a publication based on multiple experiments or from several publications, respectively (Figure 5C). Within the ‘experimentDescription’ element, the ‘bibref’ element refers to the relevant publication from which the cooperativity was inferred (Figure 5C). This avoids indistinguishable grouping of evidence for the single interactions and their cooperative behaviour.

This annotation approach is different from the procedures currently used to report interaction data within the PSI-MI consortium. Annotation of a molecular interaction entry is traditionally based on a single publication that describes one or more experiments in which the interactions of the entry were determined. In contrast, for cooperative interactions, we support annotation of molecular interaction mechanisms based on inference from a combination of different experimental approaches described in a single publication or even assembled by curators based on multiple publications. These approaches are not mutually exclusive and complement each other to attain a comprehensive description of molecular interactions, focusing on different aspects and levels of complexity, as illustrated here by means of cross-referencing primary interaction data from records describing cooperativity between multiple binding events.

### Documentation, tools and applications

Clear and detailed documentation about the PSI-MI format can be found on the PSI-MI website (http://www.psidev.info/groups/molecular-interactions). A separate website was created and linked from the PSI-MI web page to explain the annotation of cooperative interactions in this format and to give an overview of the new CV terms that were defined for this purpose (http://psi-mi-cooperativeinteractions.embl.de/). The complete PSI-MI CV can either be found in the Open Biomedical Ontologies (OBO) flat file format (http://obo.cvs.sourceforge.net/viewvc/obo/obo/ontology/genomic-proteomic/protein/psi-mi.obo), which is also linked from the PSI-MI website, or can be accessed through the ontology lookup service (http://www.ebi.ac.uk/ontology-lookup/), which allows searching and browsing different ontologies (76). The new CV terms were also integrated into the web-based IntAct editorial tool (freely available at http://code.google.com/p/intact/wiki/Editor) (26), which provides a user interface for easy and efficient curation of interaction data in the PSI-MI format, hence allowing it to be used for annotation of cooperative interactions. In addition, several tools have been developed based on the PSI-MI XML schema (http://www.psidev.info/mif#tools), including the Java-based PSI Validator that can be used for syntactic and semantic validation of PSI-MI XML files and is available as a web application (http://www.ebi.ac.uk/intact/validator/start.xhtml) (77). Also, style sheets are available to convert PSI-MI XML files to HTML, making them more human-readable.

The recently developed switches.ELM resource (http://switches.elm.eu.org) (78), a database and analysis tool for switching mechanisms that regulate the functions of short linear motifs, and thus the interactions mediated by these interfaces, captures the context-dependency and interdependency of molecular interactions in great detail. Similar to the large interaction databases like IntAct (26), DIP (27) and MINT (28), switches.ELM will provide the possibility to export curated interaction data in PSI-MI format. However, switches.ELM will additionally use the newly defined CV terms to annotate interdependencies between the distinct binding events, as well as the underlying mechanisms, and as such represent a first implementation of the standard data format presented here.

## Conclusions

The large molecular ensembles that drive cellular processes are highly dynamic and can operate as deterministic signalling engines capable of regulatory decision-making. Both complex assembly and functionality is highly dependent on the cooperative nature of the interactions between the individual components. Owing to the interdependencies between multiple distinct binding events, the behaviour of the components in isolation does not reflect the behaviour of these complexes as a whole. As a result, the emergent properties of such complex biological systems and the interactions between their constituents cannot be fully characterized by only investigating individual parts outside of their molecular context, a view that is rapidly gaining support. As methodology further advances, cooperative interactions will be characterized in more and more detail at a continuously increasing rate, which will allow us to gain a better understanding of this key phenomenon and the systems that exploit it. Because the main role of bioinformatics is to extend knowledge on biological systems and forward experimental research, it cannot lag behind in acknowledging and incorporating the interdependencies between molecular interactions that are fundamental to cell regulation. Having the ability to represent and exchange cooperative interaction data in a standardized computer-readable format is a first prerequisite to achieve this integration. This will facilitate the development and use of resources to store, exchange and analyse such data, which are currently dispersed in literature.

A wide array of data exchange formats is available to capture different aspects of cell regulation and signalling at different levels of complexity (31). Well known examples include the Systems Biology Markup Language (SBML) to describe models of biological processes (79), and the Biological Pathway Exchange standard BioPAX (80). We chose the PSI-MI format to capture cooperative interaction data because this commonly used data standard provides the means to unambiguously and consistently annotate experimentally validated molecular interactions and allows annotation of molecular details such as binding sites or modifications (33). Currently, our main goal is to link interdependent interactions, each of which can be individually captured in-depth in a PSI-MI entry, and qualitatively describe their cooperative behaviour. This could act as a link between PSI-MI, describing independent interactions, and BioPAX, describing sets of interactions constituting a pathway, because these two formats were designed to be compatible. The use of SBML seemed appropriate only when the emphasis would have been on quantification of cooperative interaction models for simulation, which is beyond our scope for now and might prove difficult owing to the limited availability of quantitative cooperative interaction data and appropriate models.

We enhanced the PSI-MI format to enable it to capture cooperative interactions by defining new CV terms that can be used as interaction attributes. However, because this strategy only enables semi-structured annotation and does not allow highly detailed description of the data, especially when considering experimental and quantitative aspects, this standard is not yet ideal. Moreover, the use of CV terms as interaction attribute names to describe cooperative effects of one interaction on a subsequent interaction is difficult to validate automatically. Another issue is the occurrence of repetition and redundancy. Because only one cooperative effect can be described in the attribute list of an interaction, an interaction that has multiple effects has to be repeated for each of these. Furthermore, this strategy does not allow describing interdependencies between interactions across entries, because single binding events and their cooperative effects annotated in one entry cannot be referenced from another entry. Although cooperatively assembled complexes, considered as a single interactor, can be reused in other entries, the format is not intended to describe whole pathways. To this end, the more appropriate BioPAX standard is available (80). Ideally, as cooperativity is intrinsic to molecular interactions in biology, it should be inherent to a molecular interaction standard. As standards development is a continuous process addressing the user’s changing needs, this format is likely to alter and improve in the future. We already addressed these limitations by defining an extended PSI-MI XML schema that allows more efficient and structured annotation of cooperative interaction data. These changes can be incorporated when a new version of the PSI-MI XML schema is released. One important aspect of data representation standards, however, is their stability over time, because a wide variety of tools are available that are developed to be compatible with the current versions of the underlying schemas. Making changes to a schema implies redeveloping these tools, which is time and effort consuming. Because the strategy described here does not involve any changes to the current PSI-MI XML schema, backward compatibility is maintained. Another advantage is that it provides flexibility, as new CV terms can be defined to describe additional features of cooperative interactions, for instance, based on the classification scheme for allosteric mechanisms that has been defined previously (81) or information captured in the AlloSteric Database (ASD) (82).

In conclusion, the new CV terms introduced here, in combination with the already available PSI-MI toolkit, provide an adequate solution to describe cooperative interactions using the current version of the PSI-MI format. It allows both experimentalists and bioinformaticians to capture, exchange and analyse cooperative interaction data. Integration of these terms into the previously developed IntAct editorial tool facilitates curation of such complex interaction data (26). The format can be used to import these data into existing interaction databases, but also serve as a basis for new databases that aim to explicitly capture the interdependencies between molecular interactions. It also allows export of cooperative interaction data, for instance, for analysis and visualization with the PSI-MI–compliant Cytoscape software (37). Moreover, it might promote the development of improved or new visualization tools that address the community need for a graphical interface capable of producing a more appropriate rendition of cooperative interactions and macromolecular complexes from a PSI-MI XML file compared with the current network representations. As a first implementation, data from the switches.ELM resource (78) will be made available in this enhanced PSI-MI format. In addition, this strategy might be used to, at least partially, recover interaction data from the BIND database that could not be mapped to the PSI-MI format, such as interaction interdependencies, during a recent conversion that was aimed at making the BIND data more generally accessible and compatible with current software (36). Last but not least, the availability of this format will hopefully further increase awareness of the importance of cooperative interactions for cell regulation among the scientific community and advance their comprehensive representation in bioinformatics.

## Supplementary data

Supplementary data are available at *Database* Online.

Supplementary Data
