# Supplementary material for: Capturing cooperative interactions with the PSI-MI format
Source: Database (Oxford). 2013 Sep 24;2013:bat066. doi: 10.1093/database/bat066 (PMC3782717; doi:10.1093/database/bat066)
Supplement: Supplementary Data [file supp_2013_bat066_index.html]

Supplementary Data 

# Capturing cooperative interactions with the PSI-MI format

## Supplementary Data

files

**Files in this Data Supplement:**

- Supplementary Data - html file
- Supplementary Data - xml file
