# Supplementary material for: Capturing cooperative interactions with the PSI-MI format
Source: Database (Oxford). 2013 Sep 24;2013:bat066. doi: 10.1093/database/bat066 (PMC3782717; doi:10.1093/database/bat066)
Supplement: Supplementary Data [file supp_bat066_DATABASE-2013-0006_Suppl_FigureS2.html]

DATABASE-2013-0006\_Suppl\_FigureS2 

# Capturing cooperative interactions with the PSI-MI format

|  |  |
| --- | --- |
| ExperimentList | |
| Experiment #30 | |
| Name: | cheng-2006-1 |
| pubmed | 16707497  [primary-reference] |
| Host Organism: | in vitro (taxid: -1) |
| Interaction detection method: | |  |  | | --- | --- | | Name: | inferred by author | | psi-mi | MI:0363 [identical object] | |
| Participant identification method: | |  |  | | --- | --- | | Name: | predetermined | | psi-mi | MI:0396 [identical object] | |
|
| publication year | 2006 |
| author-list | Cheng et al. |
| Experiment #31 | |
| Name: | stevenson-2002-1 |
| pubmed | 12081504  [primary-reference] |
| Host Organism: | in vitro (taxid: -1) |
| Interaction detection method: | |  |  | | --- | --- | | Name: | inferred by author | | psi-mi | MI:0363 [identical object] | |
| Participant identification method: | |  |  | | --- | --- | | Name: | predetermined | | psi-mi | MI:0396 [identical object] | |
|
| publication year | 2002 |
| author-list | Stevenson et al. |
| Experiment #32 | |
| Name: | jeffrey-1995-1 |
| pubmed | 7630397  [primary-reference] |
| Host Organism: | in vitro (taxid: -1) |
| Interaction detection method: | |  |  | | --- | --- | | Name: | inferred by author | | psi-mi | MI:0363 [identical object] | |
| Participant identification method: | |  |  | | --- | --- | | Name: | predetermined | | psi-mi | MI:0396 [identical object] | |
|
| publication year | 1995 |
| author-list | Jeffrey et al. |
| Experiment #33 | |
| Name: | Brown-1999-1 |
| pubmed | 10559988  [primary-reference] |
| Host Organism: | in vitro (taxid: -1) |
| Interaction detection method: | |  |  | | --- | --- | | Name: | inferred by author | | psi-mi | MI:0363 [identical object] | |
| Participant identification method: | |  |  | | --- | --- | | Name: | predetermined | | psi-mi | MI:0396 [identical object] | |
|
| publication year | 1999 |
| author-list | Brown et al. |
| Experiment #34 | |
| Name: | russo-1996-1 |
| pubmed | 8756328  [primary-reference] |
| Host Organism: | in vitro (taxid: -1) |
| Interaction detection method: | |  |  | | --- | --- | | Name: | inferred by author | | psi-mi | MI:0363 [identical object] | |
| Participant identification method: | |  |  | | --- | --- | | Name: | predetermined | | psi-mi | MI:0396 [identical object] | |
|
| publication year | 1996 |
| author-list | Russo et al. |

|  |  |
| --- | --- |
| InteractorList | |
| Interactor #1 | |
| Name: | Cdk2: Cyclin-dependent kinase 2 (gene name: CDK2) (synonym: p33 protein kinase) |
| uniprot knowledge base | P24941  [identical object] |
| refseq | NP\_001789.2  [identical object] |
| ensembl | ENSG00000123374  [gene product] |
| Interactor Type: | |  |  | | --- | --- | | Name: | protein | | psi-mi | MI:0326 [identical object] | |
| Organism: | Human |
| Interactor #2 | |
| Name: | Ccna2: Cyclin-A2 (gene name: CCNA2) (synonym: CCN1) |
| uniprot knowledge base | P20248  [identical object] |
| refseq | NP\_001228.1  [identical object] |
| ensembl | ENSG00000145386  [gene product] |
| Interactor Type: | |  |  | | --- | --- | | Name: | protein | | psi-mi | MI:0326 [identical object] | |
| Organism: | Human |
| Interactor #3 | |
| Name: | Cdk7: Cyclin-dependent kinase 7 (gene name: CDK7) (synonym: CDK-activating kinase 1) |
| uniprot knowledge base | P50613  [identical object] |
| refseq | NP\_001790.1  [identical object] |
| ensembl | ENSG00000134058  [gene product] |
| Interactor Type: | |  |  | | --- | --- | | Name: | protein | | psi-mi | MI:0326 [identical object] | |
| Organism: | Human |
| Interactor #4 | |
| Name: | Cdc6: Cell division control protein 6 homolog (gene name: CDC6) (synonym: Cdc18-related protein) |
| uniprot knowledge base | Q99741 |
| refseq | NP\_001245.1  [identical object] |
| ensembl | ENSG00000094804  [gene product] |
| Interactor Type: | |  |  | | --- | --- | | Name: | protein | | psi-mi | MI:0326 [identical object] | |
| Organism: | Human |

|  |  |
| --- | --- |
| InteractionList | |
| Interaction #5 | |
| Name: | CyclinA-Cdk2 |
| wwpdb | 1FIN  [identical object] |
| intact | EBI-1030422  [identical object] |
| Experiments: | cheng-2006-1 |
| Participants: |  |
| Participant #13 | |  |  | | --- | --- | | Cdk2 | | | Biological Role: | |  |  | | --- | --- | | Name: | unspecified role | | psi-mi | MI:0499 [identical object] | | | Experimental Role: | |  |  | | --- | --- | | Name: | unspecified role | | psi-mi | MI:0499 [identical object] | | | | |
| Participant #14 | |  |  | | --- | --- | | Ccna2 | | | Biological Role: | |  |  | | --- | --- | | Name: | unspecified role | | psi-mi | MI:0499 [identical object] | | | Experimental Role: | |  |  | | --- | --- | | Name: | unspecified role | | psi-mi | MI:0499 [identical object] | | | | |
| Interaction Type: | |  |  | | --- | --- | | Name: | direct interaction | | psi-mi | MI:0407 | |
| Interaction attributes: |  |
| cooperative mechanism | pre-assembly |
| affected interaction | CyclinA\_pCdk2-Cdc6 |
| cooperative effect outcome | positive cooperative effect |
| pre-assembly response | configurational pre-organization |
| Interaction #6 | |
| Name: | CyclinA-Cdk2 |
| wwpdb | 1FIN  [identical object] |
| intact | EBI-1030422  [identical object] |
| Experiments: | stevenson-2002-1 jeffrey-1995-1 |
| Participants: |  |
| Participant #15 | |  |  | | --- | --- | | Cdk2 | | | Biological Role: | |  |  | | --- | --- | | Name: | allosteric molecule | | psi-mi | MI:1159 [identical object] | | | Experimental Role: | |  |  | | --- | --- | | Name: | unspecified role | | psi-mi | MI:0499 [identical object] | | | | |
| Participant #16 | |  |  | | --- | --- | | Ccna2 | | | Biological Role: | |  |  | | --- | --- | | Name: | allosteric effector | | psi-mi | MI:1160 [identical object] | | | Experimental Role: | |  |  | | --- | --- | | Name: | unspecified role | | psi-mi | MI:0499 [identical object] | | | | |
| Interaction Type: | |  |  | | --- | --- | | Name: | direct interaction | | psi-mi | MI:0407 | |
| Interaction attributes: |  |
| cooperative mechanism | allostery |
| affected interaction | CyclinA\_pCdk2-Cdc6 |
| cooperative effect outcome | positive cooperative effect |
| allosteric molecule | Participant#15 |
| allosteric effector | Participant#16 |
| allosteric mechanism | allosteric change in structure |
| allosteric response | allosteric v-type response |
| allostery type | heterotropic allostery |
| Interaction #7 | |
| Name: | CyclinA-Cdk2 |
| wwpdb | 1FIN  [identical object] |
| intact | EBI-1030422  [identical object] |
| Experiments: | stevenson-2002-1 jeffrey-1995-1 |
| Participants: |  |
| Participant #17 | |  |  | | --- | --- | | Cdk2 | | | Biological Role: | |  |  | | --- | --- | | Name: | allosteric molecule | | psi-mi | MI:1159 [identical object] | | | Experimental Role: | |  |  | | --- | --- | | Name: | unspecified role | | psi-mi | MI:0499 [identical object] | | | | |
| Participant #18 | |  |  | | --- | --- | | Ccna2 | | | Biological Role: | |  |  | | --- | --- | | Name: | allosteric effector | | psi-mi | MI:1160 [identical object] | | | Experimental Role: | |  |  | | --- | --- | | Name: | unspecified role | | psi-mi | MI:0499 [identical object] | | | | |
| Interaction Type: | |  |  | | --- | --- | | Name: | direct interaction | | psi-mi | MI:0407 | |
| Interaction attributes: |  |
| cooperative mechanism | allostery |
| affected interaction | CyclinA\_pCdk2-Cdc6 |
| cooperative effect outcome | positive cooperative effect |
| allosteric molecule | Participant#17 |
| allosteric effector | Participant#18 |
| allosteric mechanism | allosteric change in structure |
| allosteric response | allosteric k-type response |
| allostery type | heterotropic allostery |
| Interaction #8 | |
| Name: | CyclinA-Cdk2 |
| wwpdb | 1FIN  [identical object] |
| intact | EBI-1030422  [identical object] |
| Experiments: | jeffrey-1995-1 |
| Participants: |  |
| Participant #19 | |  |  | | --- | --- | | Cdk2 | | | Biological Role: | |  |  | | --- | --- | | Name: | allosteric molecule | | psi-mi | MI:1159 [identical object] | | | Experimental Role: | |  |  | | --- | --- | | Name: | unspecified role | | psi-mi | MI:0499 [identical object] | | | | |
| Participant #20 | |  |  | | --- | --- | | Ccna2 | | | Biological Role: | |  |  | | --- | --- | | Name: | allosteric effector | | psi-mi | MI:1160 [identical object] | | | Experimental Role: | |  |  | | --- | --- | | Name: | unspecified role | | psi-mi | MI:0499 [identical object] | | | | |
| Interaction Type: | |  |  | | --- | --- | | Name: | direct interaction | | psi-mi | MI:0407 | |
| Interaction attributes: |  |
| cooperative mechanism | allostery |
| affected interaction | CyclinA\_Cdk2-Cdk7 |
| affected interaction | CyclinA\_Cdk2-Cdk7 |
| affected interaction | CyclinA\_Cdk2-Cdk7 |
| cooperative effect outcome | positive cooperative effect |
| allosteric molecule | Participant#19 |
| allosteric effector | Participant#20 |
| allosteric mechanism | allosteric change in structure |
| allosteric response | allosteric k-type response |
| allostery type | heterotropic allostery |
| Interaction #9 | |
| Name: | CyclinA\_Cdk2-Cdk7 |
| wwpdb | 1JST  [identical object] |
| dip | DIP57013E  [identical object] |
| Experiments: | stevenson-2002-1 Brown-1999-1 |
| Participants: |  |
| Participant #21 | |  |  | | --- | --- | | CyclinA-Cdk2 | | | Biological Role: | |  |  | | --- | --- | | Name: | enzyme target | | psi-mi | MI:0502 [identical object] | | | Experimental Role: | |  |  | | --- | --- | | Name: | unspecified role | | psi-mi | MI:0499 [identical object] | | | Feature #50 | phosphorylated residue () [ 160 .. 160 ] | | resulting-ptm |  | | participant-ref | Participant#13 | | | |
| Participant #22 | |  |  | | --- | --- | | Cdk7 | | | Biological Role: | |  |  | | --- | --- | | Name: | enzyme | | psi-mi | MI:0501 [identical object] | | | Experimental Role: | |  |  | | --- | --- | | Name: | unspecified role | | psi-mi | MI:0499 [identical object] | | | | |
| Interaction Type: | |  |  | | --- | --- | | Name: | phosphorylation reaction | | psi-mi | MI:0217 | |
| Interaction attributes: |  |
| cooperative mechanism | allostery |
| affected interaction | CyclinA\_pCdk2-Cdc6 |
| cooperative effect outcome | positive cooperative effect |
| allosteric molecule | Participant#13 |
| allosteric post-translational modification | Feature#50 |
| allosteric mechanism | allosteric change in structure |
| allosteric response | allosteric k-type response |
| allostery type | heterotropic allostery |
| Interaction #10 | |
| Name: | CyclinA\_Cdk2-Cdk7 |
| wwpdb | 1JST  [identical object] |
| dip | DIP57013E  [identical object] |
| Experiments: | stevenson-2002-1 russo-1996-1 |
| Participants: |  |
| Participant #23 | |  |  | | --- | --- | | CyclinA-Cdk2 | | | Biological Role: | |  |  | | --- | --- | | Name: | enzyme target | | psi-mi | MI:0502 [identical object] | | | Experimental Role: | |  |  | | --- | --- | | Name: | unspecified role | | psi-mi | MI:0499 [identical object] | | | Feature #51 | phosphorylated residue () [ 160 .. 160 ] | | resulting-ptm |  | | participant-ref | Participant#13 | | | |
| Participant #24 | |  |  | | --- | --- | | Cdk7 | | | Biological Role: | |  |  | | --- | --- | | Name: | enzyme | | psi-mi | MI:0501 [identical object] | | | Experimental Role: | |  |  | | --- | --- | | Name: | unspecified role | | psi-mi | MI:0499 [identical object] | | | | |
| Interaction Type: | |  |  | | --- | --- | | Name: | phosphorylation reaction | | psi-mi | MI:0217 | |
| Interaction attributes: |  |
| cooperative mechanism | allostery |
| affected interaction | CyclinA\_pCdk2-Cdc6 |
| cooperative effect outcome | positive cooperative effect |
| allosteric molecule | Participant#13 |
| allosteric post-translational modification | Feature#51 |
| allosteric mechanism | allosteric change in structure |
| allosteric response | allosteric v-type response |
| allostery type | heterotropic allostery |
| Interaction #11 | |
| Name: | CyclinA\_Cdk2-Cdk7 |
| wwpdb | 1JST  [identical object] |
| dip | DIP57013E  [identical object] |
| Experiments: | cheng-2006-1 Brown-1999-1 |
| Participants: |  |
| Participant #25 | |  |  | | --- | --- | | CyclinA-Cdk2 | | | Biological Role: | |  |  | | --- | --- | | Name: | enzyme target | | psi-mi | MI:0502 [identical object] | | | Experimental Role: | |  |  | | --- | --- | | Name: | unspecified role | | psi-mi | MI:0499 [identical object] | | | Feature #52 | phosphorylated residue () [ 160 .. 160 ] | | resulting-ptm |  | | participant-ref | Participant#13 | | | |
| Participant #26 | |  |  | | --- | --- | | Cdk7 | | | Biological Role: | |  |  | | --- | --- | | Name: | enzyme | | psi-mi | MI:0501 [identical object] | | | Experimental Role: | |  |  | | --- | --- | | Name: | unspecified role | | psi-mi | MI:0499 [identical object] | | | | |
| Interaction Type: | |  |  | | --- | --- | | Name: | phosphorylation reaction | | psi-mi | MI:0217 [identical object] | |
| Interaction attributes: |  |
| cooperative mechanism | pre-assembly |
| affected interaction | CyclinA\_pCdk2-Cdc6 |
| cooperative effect outcome | positive cooperative effect |
| pre-assembly response | altered physicochemical compatibility |
| Interaction #12 | |
| Name: | CyclinA\_pCdk2-Cdc6 |
| wwpdb | 2CCI  [identical object] |
| Experiments: | cheng-2006-1 |
| Participants: |  |
| Participant #27 | |  |  | | --- | --- | | CyclinA-Cdk2 | | | Biological Role: | |  |  | | --- | --- | | Name: | enzyme | | psi-mi | MI:0501 [identical object] | | | Experimental Role: | |  |  | | --- | --- | | Name: | unspecified role | | psi-mi | MI:0499 [identical object] | | | Feature #53 | phosphorylated residue () [ 160 .. 160 ] | | prerequisite-ptm |  | | participant-ref | Participant#13 | | | |
| Participant #28 | |  |  | | --- | --- | | Cdc6 | | | Biological Role: | |  |  | | --- | --- | | Name: | enzyme target | | psi-mi | MI:0502 [identical object] | | | Experimental Role: | |  |  | | --- | --- | | Name: | unspecified role | | psi-mi | MI:0499 [identical object] | | | Feature #54 | phosphorylated residue () [ 74 .. 74 ] | | resulting-ptm |  | | | |
| Interaction Type: | |  |  | | --- | --- | | Name: | phosphorylation reaction | | psi-mi | MI:0217 [identical object] | |
